# Measuring Reasoning in Paranoia: Development of the Fast and Slow Thinking Questionnaire

**DOI:** 10.1093/schizbullopen/sgaa035

**Published:** 2020-07-10

**Authors:** Amy Hardy, Eva Tolmeijer, Victoria Edwards, Thomas Ward, Daniel Freeman, Richard Emsley, Catherine Green, Maria Rus-Calafell, Kathryn Greenwood, Paul Bebbington, Elizabeth Kuipers, David Fowler, Catarina Sacadura, Nicola Collett, Alison McGourty, Graham Dunn, Philippa Garety

**Affiliations:** 1 Department of Psychology, Institute of Psychiatry, Psychology & Neuroscience, King’s College London, De Crespigny Park, UK; 2 South London & Maudsley NHS Foundation Trust, Bethlem Royal Hospital, Beckenham, Kent, UK; 3 Department of Clinical Psychology, VU University and Amsterdam Public Health Research, Amsterdam, The Netherlands; 4 Parnassia Psychiatric Institute, The Hague, Netherlands; 5 Department of Psychiatry, University of Oxford, Oxford, UK; 6 Oxford Health NHS Foundation Trust, Oxford, UK; 7 Department of Biostatistics and Health Informatics, Institute of Psychiatry, Psychology & Neuroscience, King’s College London, London, UK; 8 Mental Health Research and Treatment Centre, Clinical Child and Adolescent Psychology, Ruhr-Universität Bochum, Bochum, Germany; 9 Department of Psychology, University of Sussex, Sussex, UK; 10 Sussex Partnership NHS Foundation Trust, Sussex, UK; 11 UCL Mental Health Sciences Unit, University College London, London, UK; 12 City, University of London, London, UK; 13 School of Health Sciences, University of Manchester, Manchester, UK

**Keywords:** cognitive biases, jumping to conclusions, belief flexibility, assessment, psychosis, paranoid thoughts, paranoia, schizophrenia, questionnaire

## Abstract

Paranoid thoughts are common across the psychosis continuum. It is well established that reasoning biases (conceived as an overreliance on fast thinking and lack of willingness and/or ability to engage in slow thinking) contribute to paranoia. Targeted therapies have shown promise in improving reasoning in order to reduce paranoia. Psychometrically robust and easy-to-use measures of these thinking styles will assist research and clinical practice. Existing assessments include experimental tasks that are complex to administer or self-report measures that have limitations in comprehensively assessing cognitive biases in paranoia. We have developed the first questionnaire to assess fast and slow thinking biases related to paranoid thoughts, and here report on its evaluation. In study 1, we generated, evaluated, and extracted items reflecting reasoning, and assessed their reliability and validity in a non-clinical sample (*n* = 209). In study 2, we replicated the factor analysis and psychometric evaluation in a clinical sample (*n* = 265). The resultant *Fast and Slow Thinking* (FaST) questionnaire consists of two 5-item scales reflecting fast and slow thinking and is therefore brief and suitable for use in both research and clinical practice. The fast thinking scale is reliable and valid. Reliability and criterion validity of the slow scale shows promise. It had limited construct validity with objective reasoning assessments in the clinical group, possibly due to impaired meta-cognitive awareness of slow thinking. We recommend the FaST questionnaire as a new tool for improving understanding of reasoning biases in paranoia and supporting targeted psychological therapies.

## Introduction

“If I think fast, I know everyone is against me, I feel so sure. Now I try not to jump to conclusions, I slow down, take a step back and consider whether people are just doing their own thing.” (Quote from a participant in the SlowMo Trial^1^).

Paranoid and suspicious thoughts, or fear of harm from others, are common in the general population, and occur in a range of mental health problems.^2,3^ They cause significant distress and functional impairment, and treatment innovations are needed to improve outcomes.^4^ Theoretical models have highlighted the role of cognitive, behavioral and emotional processes in paranoia, while psychological interventions specifically targeting these mechanisms show promise.^5–8^ Psychometrically robust and easy-to-use assessment measures of these empirically identified mechanisms will assist research and clinical practice. A number of standardized self-report measures exist, providing reliable and efficient assessment methods for a range of the key putative factors (eg, for sleep disturbance,^9^ excessive worry,^10^ low self-confidence,^11^ intolerance of anxiety,^12^ anomalous experiences,^13^ safety or defense behaviors.^14^ However, there is no such tool for the specific reasoning biases (as outlined in the above quote) that may exacerbate or ameliorate paranoia. We have developed and evaluated the first questionnaire for assessing reasoning biases related to paranoid thoughts.

In our work, we have conceptualized the thinking styles associated with paranoia within dual processing models of reasoning.^15–18^ Kahneman notably coined the phrase, “Thinking, fast and slow,” in a best-selling book of this title, to describe the systematic thinking errors or biases used by humans in making decisions and judgments. *Fast* or experiential (“type 1”) reasoning reflects emotion-driven, instinctive thinking processes. In contrast, *slow* or analytic (“type 2”) reasoning consists of reflective and rational thinking processes and is dependent on cognitive capacity and functioning.

Evidence suggests that the reasoning biases associated with paranoia can be framed as an overreliance on fast thinking coupled with insufficient willingness and/or ability to engage in slow thinking.^18,19^ For example, jumping to conclusions (JTC) bias is the tendency to use less data to reach a conclusion, such that appraisals of anomalous or ambiguous information are drawn on the basis of reduced information gathering.^20^ Robust evidence demonstrates JTC is 4 and 6 times more common in people with psychosis than in non-clinical and non-psychotic samples, respectively and is specifically associated with delusions.^21–24^ Belief flexibility (BF) involves the willingness and ability to take a step back from one’s own beliefs, to reflect and modify them in line with newer information, and to generate and consider alternative explanations.^20,25^ These processes are derived in part from hierarchical Bayesian models of reasoning, whereby faulty prediction errors reflect belief inflexibility, giving rise to psychotic symptoms, including paranoia.^26,27^ In psychosis research, BF is assessed according to whether people can recognize the “possibility of being mistaken” (PM) about their beliefs and whether they have any “alternative explanations” (AEs) for their experiences. A lack of BF is common in people with delusions and psychosis, with only 50% reporting the possibility of being mistaken and a quarter reporting AEs.^20,28,29^ A related construct is the “Bias against Disconfirmatory Evidence” (BADE), in which disconfirmatory evidence is neglected.^30^ BADE is also associated with delusions, and together with JTC, may be linked to difficulties with the integration of both confirmatory and disconfirmatory evidence.^23,31^

To date, the constructs related to fast and slow thinking in paranoia are usually assessed by experimental, performance measures (ie, the beads task for JTC^25^ and the BADE task,^30^ clinical research interviews (ie, the Maudsley Assessment of Delusions, MADS, for PM^32^ and the Explanation of Experiences, EoE,^28^ for AEs), general self-report questionnaires (eg, Rational Experiential Inventory, REI^33^) and psychosis-specific self-report questionnaires (eg, Beck Cognitive Insight Scale, BCIS^34^; Cognitive Biases Questionnaire, CBQ,^35^ Davos Assessment of Cognitive Biases Scale, DACOBS^36^). While there are helpful self-report measures for assessing reasoning in psychosis, these do not specifically target reasoning biases in relation to paranoia. Further, there is currently no measure that meets all key psychometric properties (ie, internal consistency, test-rest reliability, construct validity, criterion validity) for assessing paranoia-related reasoning biases.

The CBQ is not associated with the beads task or MADS and is better viewed as measuring interpretation biases.^35^ The DACOBS JTC and BF subscales have moderate associations with the beads (60:40 ratio) task, but they have not been explored in relation to other empirically established reasoning biases (ie, PM and AEs) and there are equivocal findings regarding their relationship to paranoia.^37,38^ The BCIS has not been validated with paranoia-specific reasoning tasks, and its construct validity has only been assessed in relation to delusions, not paranoia.^39^ The REI does not appear to tap into paranoia-specific reasoning, as people with psychosis (*n* = 30) reported lower levels of both experiential (fast) and rational (slow) thinking compared to a non-clinical group, and experiential thinking was not associated with paranoia in either group (*n* = 1000).^19^ We identified a need for a self-report questionnaire that accurately and concisely measures the key reasoning biases relevant to paranoia and can be used across the psychosis continuum. This would provide an easy-to-use assessment, and overcome the limitations of experimental tasks and interviews that are complex to administer, subject to biases in administration, specific to a single, paranoid belief, or not specific to paranoia at all. Consequently, we developed a measure for assessing self-reported reasoning in paranoia, the *Fast and Slow Thinking* (FaST) questionnaire.

A challenge inherent to self-report assessments is that their validity depends on people’s self-awareness, and responses may not reflect objective performance. Awareness of one’s own thinking processes is a meta-cognitive process and it has long been recognized accurate reflections are limited in the general population, particularly in the context of fast thinking.^40^ Impairments in this area may impede the utility of self-report questionnaires, especially if difficulties with self-awareness are greater in people with clinically significant paranoia. To address this concern, we validated the FaST in both non-clinical and clinical samples and assessed its construct validity in relation to performance tasks and interview assessments of reasoning.

We conducted 2 studies. In study 1, we generated, evaluated, and extracted items reflecting reasoning across the continuum of paranoia. Then we assessed their reliability and validity by examining internal consistency, test-retest reliability, construct validity, and criterion validity in a non-clinical sample. In study 2, we further investigated the questionnaire by replicating the factor analysis and validity evaluations in a clinical sample. Notwithstanding the potential meta-cognitive awareness limitations discussed above, we predicted that self-reported fast and slow thinking would, respectively, be positively and negatively associated with paranoia, JTC, and belief inflexibility (PM and AEs). We anticipated that clinical participants would have significantly higher fast and lower slow thinking scores than the non-clinical group.

## Study One: Non-clinical Group

### Method

#### Item Pool Construction.

Fifty-five items formed the initial pool. Items were selected from reviewing existing measures of belief and cognitive flexibility and through expert consultation. The measures comprised the REI, the MADS, and the EoE. The item pool included 33 items involving perceived engagement with fast and slow thinking, and 22 items involving perceived ability and willingness to use the 2 reasoning styles. Perceived engagement was subdivided into items reflecting the ability to generate alternative explanations and to gather information, disconfirmatory processing, and the possibility of being mistaken. No items created were identical to those in existing measures, although some were similar in content. Respondents were prompted to indicate the extent to which they agreed with each of the statements when they had a paranoid or suspicious thought. Items were scored on a 5-point Likert scale from 1 = “not at all” to 5 = “totally”.

### Participants

The sample was recruited by circular email to people working or studying at King’s College London. Two hundred nine participants completed the FaST questionnaire and a measure of paranoia, Green Paranoid Thought Scale (GPTS^41^). They ranged in age from 17 to 62 years (mean = 26.4, SD = 7.7). The majority of the sample were female (69%), white (41%), and full-time students (61%). The 136 individuals who did not complete both the FaST and the GPTS (*n* = 136) were excluded from the analysis. They did not differ significantly in age, sex, or employment status from those who were included.

### Procedure

All participants were administered the pool of 55 items and other self-report measures, including the GPTS and the REI to assess criterion and construct validity at baseline (T1). The GPTS is a 32-item self-report measure of levels of paranoia. It consists of two 16-item subscales relating to ideas of social reference and persecution. Items are rated on a 5-point Likert scale, with higher scores indicating greater severity. The GPTS has been evaluated in non-clinical and clinical populations and has good internal consistency, validity, and test-retest reliability.^41^ The REI is a 40-item self-report scale that distinguishes between rational (comparable to slow thinking) and experiential (comparable to fast thinking) cognitive styles. Each cognitive style has an engagement and ability subscale. Items are rated on a 5-point Likert scale, with higher scores indicating greater ability and engagement. Reliability and validity have been demonstrated in non-clinical samples. Participants completed assessments online using survey software, SurveyMonkey. An information sheet was provided and informed consent was obtained. A subsample of participants who consented to be re-contacted were emailed 2 weeks after baseline (T2) and asked to complete the item pool and GPTS again.

### Analysis

The 55 items were examined for endorsement before being subjected to factor analysis using varimax rotation. Following this, items were reconsidered for final inclusion. Reliability of the final items was assessed using internal consistency and test-retest. Construct and criterion validity was also investigated.

## Results

### FaST Questionnaire Item Extraction

No items had an endorsement below 10% (>90% of responses at the extreme). The Cronbach’s alpha coefficient of the 55 items was found to be high (α = 0.92), with all items above 0.92 on Cronbach’s Alpha if item deleted. Item scores were submitted to principal axis factoring with a scree plot. Data were suitable for principal axis factoring, as demonstrated by 0.89 on the Kaiser-Meyer-Olkin measure of sampling adequacy and a significant Bartlett’s test of sphericity (χ ^2^ (1485) = 8587.4, *P* < .0001).

Following Cattell’s^42^ criterion, the scree plot indicated the data were best described by 2 components that explained 43.6% of the sample variance. Two factors were extracted using varimax rotation. Factor 1 comprised 31 items and explained 26.9% of the sample variance. All items related to slow thinking loaded onto factor 1 (factor loading > 0.4), which we accordingly defined as the slow thinking scale. Factor 2 comprised 23 items and explained 16.7% of the sample variance. All items related to fast thinking loaded onto factor 2 (factor loading >0.4), which was labeled as the fast thinking scale.

From the principal axis factoring results, the fast and slow scales were derived. Items were selected considering the face validity of items, the need to reflect the different components of reasoning relevant to paranoid thoughts (ie, alternative explanations, information gathering, disconfirmatory processing, and possibility of being mistaken), factor loading (>0.4), item-scale correlation and variance and endorsement. See supplementary table 1 for the item factor loadings.

### Scales and Associated Norms

Following the above criteria, 12 items were selected, 6 measuring slow thinking, and 6 reflecting fast thinking. The slow thinking scale ranged from 6 to 30 with higher scores reflecting slower thinking. The mean total score was 22.3 (range = 8.0–30.0, SD = 4.7). The fast thinking scale ranged from 6 to 30 with higher scores reflecting faster thinking. The mean total score was 12.9 (range 6.0–30.0, SD = 5.0).

### Questionnaire Reliability

The Cronbach’s α value was 0.85 for the slow thinking scale and 0.87 for the fast thinking scale, indicating adequate internal consistency. There were no significant differences between those who did and did not complete the follow-up questionnaire age, gender, ethnicity, or employment status (*P* > .05). The correlation coefficient (ICC) between scores at baseline and follow-up were significant (*P* < .001, *n* = 94). The ICC was 0.89 for slow thinking scale and 0.90 for the fast thinking scale.

### Questionnaire Validity

Criterion and construct validity was assessed by investigating the relationship between the fast and slow scales and the GPTS and REI (table 1). The fast scale had significant, medium-large positive correlations with the GPTS. Scores on the slow thinking scale had significant, small negative correlations with the GPTS ideas of reference. For the REI, the rational ability and rational engagement subscales both had medium, positive correlations with the slow thinking scale, and medium, negative correlations with the fast thinking scale. The experiential ability and experiential engagement subscales both had medium, positive correlations with the fast thinking scale, while the experiential ability subscale had a small, negative correlation with the slow thinking scale.

**Table 1.** Associations Between the 12-Item FaST Fast and Slow Scales and the GPTS in the Non-Clinical Sample

	FaST^FAST^ (*n* = 209)	FaST^SLOW^ (*n* = 209)	GPTS^TOTAL^ (*n* = 199)	GPTS^REF^ (*n* = 202)	GPTS^PERS^ (*n* = 203)	REI^RA^ (*n* = 191)	REI^RE^ (*n* = 188)	REI^EA^ (*n* = 190)	REI^EE^ (*n* = 186)
FaST^FAST^	1								
FaST^SLOW^	−.21**	1							
GPTS^TOTAL^	.40**	−.12	1						
GPTS^REF^	.35**	−.14*	.93**	1					
GPTS^PERS^	.35**	−.05	.89**	.67**	1				
REI^RA^	−.31**	.26**	−.28**	−.23**	−.29**	1			
REI^RE^	−.36**	.31**	−.20**	−.19*	−.17*	.60**	1		
REI^EA^	.43**	−.19**	.06	.06	.06	−.10	−.17*	1	
REI^EE^	.33**	−.09	.06	.02	.10	−.21**	−.11	.69**	1

*Note*: FaST, *Fast and Slow Thinking*; GPTS, Green Paranoid Thought Scale; REI, Rational Experiential Inventory.

*P < .05. **P < .01.

## Study 2: Clinical Group

### Method

#### Participants.

The sample represented a subset of participants from the SlowMo therapy trial (a multicenter RCT of a blended digital therapy for paranoia, see ISRCTN32448671^1^). Two hundred sixty-five participants consented to participate and provided data at the trial baseline assessment. All had a clinical diagnosis of psychosis (F20-F29) and all were assessed as holding current paranoid (delusional) beliefs, as assessed by the GPTS (score ≥ 29 on part B, persecutory subscale). The 265 individuals who returned completed data ranged in age from 19 to 73 years (mean = 42.4, SD = 11.8). The majority were male (69.1%), White British (71%), single (77%), and unemployed (81%). Individuals who did not provide complete GPTS and FaST data (*n* = 9) were excluded.

### Procedure

All participants were administered the 12-item questionnaire and other measures and tasks. These included: the GTPS; the persecutory delusions item of the Scales for Assessment of Positive Symptoms (SAPS^43^); the delusions scale of the Psychotic Symptom Ratings Scales^44^; the MADS (a standardized interview that assesses a person’s main delusional belief, including conviction, possibility of being mistaken, distress and preoccupation of this belief on a scale of 0–100); the EoE (a structured interview that assesses whether participants can provide alternative explanations for their main paranoid belief, with a binary [“yes” or “no” rating]); and the beads task to assess JTC. Two jars with different proportions of colored beads (85:15 or 60:40) are presented and participants are told one jar has been chosen. A sequence of beads being drawn from one of the 2 jars is shown. After each draw, participants are asked if they want to see another bead or whether they have decided with certainty as to which jar has been chosen. JTC is rated as present if the participant decides after seeing 2 or fewer beads.^21^

### Analysis

To assess replicability of the factor structure in the clinical group, the 12 items were investigated using principal axis factoring, then assessed for reliability and validity. Reliability was assessed by examining internal consistency. Construct validity was assessed by evaluating differences in scale scores between the clinical and non-clinical groups, and by correlating the questionnaire with the MADS and draws to decision on the JTC beads task, and conducting independent *t*-tests between the fast and slow thinking scales and dichotomized measures of reasoning: MADS, EoE, and JTC beads task. Criterion validity was assessed by estimating the correlation between the questionnaire and the GPTS, persecutory delusions item of the SAPS, and the delusions total of the Psychotic Symptom Rating Scales (PSYRATS).

## Results

### Questionnaire Replication

No items had an endorsement below 10% (>90% of responses at the extreme). Item scores (*n* = 12) were submitted to principal axis factoring analysis with a scree plot. Employing Cattell’s^42^ criterion, the scree plot indicated the data were best described by 2 components, explaining 48.2% of the sample variance. Two factors were extracted using varimax rotation. Factor 1 comprised 6 items (factor loading >0.4) and explained 26.6% of the sample variance. All fast thinking items were included in factor 1, thereby replicating the fast thinking scale in the non-clinical group. Factor 2 (factor loading > 0.4) comprised 5 items and explained 21.6% of the sample variance. All slow thinking items were included in factor 2, replicating the slow thinking scale in the non-clinical group. To ensure an equal number of items for each scale, one item from the fast scale was removed based on the smallest factor loading. See supplementary table 1 for the item factor loadings. The mean total score for the slow thinking scale for the non-clinical group (*n* = 209) and clinical group (*n* = 265) was 18.9 (range = 7.0–25.0, SD = 4.2) and 17.0 (range = 5.0–25.0, SD = 4.4), respectively. The mean score for the fast thinking scale for non-clinical and clinical group was 10.6 (range = 5.0–25.0, SD = 4.3) and 16.9 (range = 6.0–25.0, SD = 4.5), respectively. See the supplementary table 2, for the 10-item scale.

### Reliability

Cronbach’s α 0.77 for both scales (*n* = 265), indicating adequate internal consistency. Test-retest reliability was re-assessed in the non-clinical group for the 10-item FaST questionnaire, with consistent findings (supplementary material).

### Validity

The fast thinking scale had significant small-medium positive correlations with scores on MADS conviction, distress, and preoccupation (table 2). In contrast, the slow scale showed no significant associations. In both groups, the fast scale had significant, medium-large positive correlations with the GPTS. Scores on the slow thinking scale had small, positive, and negative correlations with the GPTS for the clinical and non-clinical group, respectively. There were also significant, small–medium correlations between fast scale and the persecutory delusions item on the SAPS and PSYRATS delusions total score. There were no significant associations between these measures and the slow scale.

**Table 2.** Associations Between the 10-Item FaST Questionnaire, GPTS, MADS, SAPS, PSYRATS, and JTC Task Draws to Decision

Non-clinical Group (*n* = 209)						Clinical Group (*n* = 265)				
	FaST^SLOW^	FaST^FAST^	GPTS^TOTAL^	GPTS^REF^	GPTS^PERS^	FaST^SLOW^	FaST^FAST^	GPTS^TOTAL^	GPTS^REF^	GPTS^PERS^
FaST^SLOW^		1				1				
FaST^FAST^	1	−.16**				−.05	1			
GPTS^TOTAL^	−.16*	.40**	1			.16*	.44**	1		
GPTS^REF^	−.16*	.36**	.93**	1		.16*	.34**	.91**	1	
GPTS^PERS^	−.10	.34**	.90**	.68**	1	.13*	.42**	.88**	.61**	1
MADS^CONV^						.11	.33**	.10	.06	.09
MADS^DIST^						.05	.23**	.09	.06	.06
MADS^PREO^						.09	.27**	.18**	.10	.15*
SAPS^PERS^						.08	.18**	.40**	.32**	.42**
PSYR^TOTAL^						.06	.34**	.51**	.42**	.51**
JTC^60:40^						−.04	−.18**	−.05	−.01	−.08
JTC^85:15^						−.06	−.10	−.01	.02	−.05

*Note*: FaST, *Fast and Slow Thinking*; GPTS, Green Paranoid Thought Scale; JTS, jumping to conclusions; MADS, Maudsley Assessment of Delusions; PSYRATS, Psychotic Symptom Rating Scales; REI, Rational Experiential Inventory; SAPS, Scales for Assessment of Positive Symptoms.

*P< .05. **P < .01.

Independent *t*-tests were conducted to compare scale scores between (1) people who indicated they could and could not be mistaken about their upsetting belief on the MADS, (2) people who did or did not have an alternative explanation for their upsetting belief on the EoE, and (3) people who did (≤2 beads) or did not jump to conclusions on the beads task (table 3). Independent *t*-tests revealed a significant difference in the mean scores on the fast thinking scale for all measures except the JTC 85:15 task. There were no significant differences on the slow thinking scale. The same results with respect to fast and slow thinking were also found for “draws to decision” on the beads tasks (table 2).

**Table 3.** Independent Samples *t*-Test for Reasoning Variables in Relation to the 10-Item FaST Questionnaire in the Clinical Group

								95% CI	
FaST Scale	Reasoning Variable	*n*	Mean	SD	*t*	df	Mean Difference	Lower Bound	Upper Bound
FaST^SLOW^	MADS^MIST^ no	104	17.3	4.6					
	MADS^MIST^ yes	161	16.8	4.3	1.0	263	0.6	0.6	−0.5
FaST^FAST^	MADS^MIST^ no	104	19.0	4.3					
	MADS^MIST^ yes	161	15.5	4.2	6.5**	263	3.5	2.4	4.5
FaST^SLOW^	EOE: no AE	138	19.5	4.9					
	EOE: AE	126	19.7	4.6	−0.4	262	−0.2	−1.4	1.0
FaST^FAST^	EOE: no AE	138	20.8	5.4					
	EOE: AE	126	19.1	4.8	2.7*	262	1.7	0.5	2.9
FaST^SLOW^	JTC^60:40^ 0–2 beads	102	17.2	4.7					
	JTC^60:40^ > 2 beads	161	16.9	4.2	0.6	261	0.4	−0.7	1.5
FaST^FAST^	JTC^60:40^ 0–2 beads	102	17.9	4.7					
	JTC^60:40^ > 2 beads	161	16.2	4.4	2.9*	261	1.6	0.5	2.7
FaST^SLOW^	JTC^85:15^ 0–2 beads	140	17.1	4.6					
	JTC^85:15^ > 2 beads	132	16.9	4.2	0.3	261	0.1	−0.9	1.2
FaST^FAST^	JTC^85:15^ 0–2 beads	140	17.3	4.8					
	JTC^85:15^ > 2 beads	132	16.4	4.3	1.5	261	0.8	−0.3	1.9

*Note*: EOE, Explanation of Experiences; FaST, *Fast and Slow Thinking*; JTS, jumping to conclusions; MADS, Maudsley Assessment of Delusions.

*P< .05. **P < .01.

Construct validity was investigated by comparing responses on the questionnaire between the clinical (*n* = 265) and non-clinical sample (*n* = 209) (table 4). For the clinical group, scores on the fast and slow thinking scales were significantly higher and lower, respectively, compared to the non-clinical group (*P* < .0001). There was nevertheless considerable overlap in the range of the scores for both scales between the non-clinical and clinical group.

**Table 4.** Comparison Between Clinical and Non-clinical Groups on the 10-Item FaST Questionnaire and the GPTS

	Mean (SD)			Range	
	Non-clinical Group (*n* = 209)	Clinical Group (*n* = 265)	*t*	Non-clinical Group (*n* = 209)	Clinical Group (*n* = 265)
FaST^FAST^	10.6 (4.3)	16.9 (4.5)***	15.3	5–25	6–25
FaST^SLOW^	18.9 (4.2)	17.0 (4.4)***	−4.9	7–25	5–25
GPTS^TOTAL^	51.8 (21.2)	106.9 (26.0)***	26.7	32–160	54–160
GPTS^REF^	29.5 (12.7)	50.1 (15.3)***	16.9	16–80	16–80
GPTS^PERS^	22.4 (10.5)	56.7 (13.7)***	32.7	16–80	30–80

*Note*: FaST, *Fast and Slow Thinking*; GPTS, Green Paranoid Thought Scale.

***P < .001.

## Discussion

The FaST questionnaire is the first brief, self-report tool, for comprehensively measuring reasoning biases in paranoia. The fast scale demonstrated test-retest reliability, internal consistency, construct validity, and criterion validity. It had positive, medium associations with paranoia severity in the clinical and non-clinical groups, experiential reasoning in the non-clinical group, and delusion severity, belief inflexibility, and JTC on one (but not both) beads tasks in the clinical group. The scale discriminated 2 groups, with the clinical group scoring significantly higher. The findings suggest that the fast scale provides a psychometrically robust assessment of fast thinking as it contributes to paranoia. This indicates that people across the psychosis continuum can accurately self-report fast thinking.

The slow thinking had adequate internal reliability and test-retest reliability, although findings in relation to construct and criterion validity were mixed. Contrary to our slow thinking hypotheses, the slow scale had a small, positive association with GPTS in the clinical group and no relation to delusions in the clinical group or persecutory ideation on the GPTS in the non-clinical group. In support of our hypotheses, the scale had small–moderate associations with rational and experiential thinking in the expected direction and had a small, negative association with GPTS delusions of reference, in the non-clinical group. The non-clinical group was also more likely to report slow thinking. Slow thinking was not associated with any MADS dimensions or reasoning variables in the clinical group. As we initially speculated, this suggests meta-cognitive awareness of slow reasoning processes may be limited, particularly in people with clinically significant paranoia. This could be exacerbated by meta-cognitive beliefs about reasoning biases, with positive beliefs about slow thinking potentially leading to elevated rates of endorsement. Nonetheless, we anticipate the slow scale will still have utility in a therapeutic context to assess, normalize, and validate slow thinking habits. It also has the potential to provide a means of framing and evaluating therapeutic work to encourage engagement of the reflective mind, through slow thinking, as illustrated below.^18^

“Slowing down shows me the jigsaw puzzle in more detail - parts which looked like they fitted together might not on closer inspection.” (Quote from a participant in the SlowMo Trial^1^).

The FaST questionnaire may perform better than existing measures for measuring paranoia-specific reasoning biases. The fast scale is robustly associated with all components of reasoning and paranoia severity, unlike the CBQ, DACOBS, REI, and BCIS, and discriminates between groups with clinically significant paranoia and those with lower levels of paranoia in a non-clinical group. While the slow thinking scale was not as psychometrically robust and may need further refinement, its addition has an advantage over the CBQ and DACOBS, as these do not assess the flexible reasoning processes that psychological therapy aims to develop. We now plan to investigate the questionnaire’s performance longitudinally: we will explore the associations between fast and slow thinking over time and their relation to therapy outcomes. We note that the fast and slow thinking scales have a small, negative association, suggesting they are not simply the inverse of each other, but separate systems and their relationship warrants further investigation. Replication is needed to investigate a larger non-clinical sample, and to address sampling biases. The non-clinical sample was fairly homogenous (predominantly female students) and the clinical sample had consented to participate in the SlowMo therapy trial, and so results may not generalize to the psychosis population as a whole. Further, different measures were used in the non-clinical and clinical samples due to the need to reduce the assessment burden in the SlowMo trial, and it not being possible to conduct interview assessments online.

In conclusion, the fast thinking scale is valid and reliable. Reliability and criterion validity of the slow scale is promising, although it showed limited construct validity in the clinical group when compared to objective reasoning assessments, possibly due to impaired meta-cognitive awareness of slow thinking. We recommend the FaST questionnaire as a new tool for improving understanding of reasoning biases in paranoia and supporting targeted psychological therapies.

## Supplementary Material

Supplementary data are available at *Schizophrenia Bulletin Open* online.

Please see supplementary materials for the FaST questionnaire. Tables 1 to 3 present the study analysis conducted with the Revised Green Paranoid Thought Scale (R-GPTS, Freeman et al, 2019), which demonstrates similar findings to the GPTS for the criterion validity of the FaST questionnaire.

sgaa035_suppl_Supplementary_Material
