# Supplementary material for: Measuring Reasoning in Paranoia: Development of the Fast and Slow Thinking Questionnaire
Source: Schizophr Bull Open. 2020 Jul 10;1(1):sgaa035. doi: 10.1093/schizbullopen/sgaa035 (PMC13701027; doi:10.1093/schizbullopen/sgaa035)
Supplement: Supplementary file 1 [file sgaa035_suppl_supplementary_material.docx]

**Supplementary material**

|  |  |  | Non-Clinical | | Clinical | |
| --- | --- | --- | --- | --- | --- | --- |
| Items | |  | FaST^FAST^ | FaST^SLOW^ | FaST^FAST^ | FaST^SLOW^ |
| 1. I rely on my gut feelings and do not look for more information | |  | 0.62 |  | 0.47 |  |
| 2. I like to logically think through what has happened. | |  |  | 0.75 |  | 0.64 |
| 3. I trust in my heart that this is definitely what is happening | |  | 0.77 |  | 0.62 |  |
| 4. I think about different viewpoints to explain what is happening | |  |  | 0.70 |  | 0.69 |
| 5. Regardless of what happens, nothing changes my mind | |  | 0.68 |  | 0.63 |  |
| 6. I like to trust my instinct or hunch about the situation | |  | 0.66 |  | 0.58 |  |
| 7. I ask myself if the thought is likely, in light of all the facts. | |  |  | 0.70 |  | 0.55 |
| 8. There is no way I am wrong about what is happening. | |  | 0.79 |  | 0.68 |  |
| 9. I look for more information about what is happening. | |  |  | 0.69 |  | 0.65 |
| 10. I think I may be mistaken about what is happening. | |  |  | 0.44 |  |  |
| 11. I know my hunch is right, and do not think of other possibilities. | |  | 0.79 |  | 0.74 |  |
| 12. I like to think about other possibilities for what is going on. | |  |  | 0.86 |  | 0.62 |

Supplementary Table 1. Items included in the 12 item FaST questionnaire and associated factor loadings (> .40) for the fast and slow scales.

Supplementary Table 2. 10 item FaST Questionnaire

**The Fast and Slow Thinking (FaST) Questionnaire**

*Please read each of the statements carefully below* and indicate the extent to which you agree with each statement, when you have a paranoid or suspicious thought.

| **When I have a paranoid or suspicious thought…..** | *Not at all* |  | *Somewhat* |  | *Totally* |
| --- | --- | --- | --- | --- | --- |
| 1. I like to logically think through what has happened. | 1 | 2 | 3 | 4 | 5 |
| 2. I trust in my heart that this is definitely what is happening. | 1 | 2 | 3 | 4 | 5 |
| 3. I think about different viewpoints to explain what is happening. | 1 | 2 | 3 | 4 | 5 |
| 4. Regardless of what happens, nothing changes my mind. | 1 | 2 | 3 | 4 | 5 |
| 5. I like to trust my instinct or hunch about the situation. | 1 | 2 | 3 | 4 | 5 |
| 6. I ask myself if the thought is likely, in light of all the facts. | 1 | 2 | 3 | 4 | 5 |
| 7. There is no way I am wrong about what is happening. | 1 | 2 | 3 | 4 | 5 |
| 8. I look for more information about what’s happening. | 1 | 2 | 3 | 4 | 5 |
| 9. I know my hunch is right, and do not think of other possibilities. | 1 | 2 | 3 | 4 | 5 |
| 10. I like to think about other possibilities for what is going on. | 1 | 2 | 3 | 4 | 5 |

Scoring:

Fast thinking: 2 + 4 + 5 + 7 + 9

Slow thinking: 1 + 3 + 6 + 8 + 10

**Test-retest reliability in non-clinical sample on the 10 item FaST questionnaire**

To assess test-retest reliability, a total of 94 individuals from the non-clinical group repeated the FaST two weeks after initial completion. The intra-class correlation coefficient (ICC) between total FaST scores at baseline and follow-up for the 10 item scale was significant (*p* < 0.001) for items relating to fast thinking (ICC = 0.88) and slow thinking (ICC = 0.89).

**Analysis of the FaST Questionnaire using the Revised Green Paranoia Thoughts Scale (r-GPTS)**

**Methods**

***Measure***

*The Revised Green et al. Paranoid Thoughts Scale – 18 items (R-GPTS; Freeman et al., 2019).* Comprises two scales assessing thinking relevant to paranoia: ideas of social reference (Part A) and persecution (Part B). The Part A scale contains 8 items and The Part B Scale contains 10 items rated over the preceding month, each scored on a five-point Likert scale from 0 (‘Not at all’) to 4 (‘Totally). Scales can be added up to form a total score. Higher scores reflect higher levels of paranoia.

**Analysis**

Criterion and construct validity were assessed by investigating the relationship between the fast and slow scales and the R-GPTS (Freeman et al., 2019) for the clinical (n=265) and non-clinical group (n=209). Construct validity was investigated by comparing responses on the questionnaire between the clinical and non-clinical group. To assess test-retest reliability, a total of 94 individuals of the non-clinical group repeated FaST two weeks after initial completion. Items for the 12 item and 10 item scale for both the clinical and non-clinical group were being subjected to factor analysis using varimax rotation.

**Results**

In both groups, the fast scale had significant, medium-large positive correlations with the R-GPTS. Scores on the slow thinking scale had small, positive and negative correlations with the R-GPTS for the clinical and non-clinical group, respectively (Supplementary Table 3 and 4). For the clinical group, scores on the fast and slow thinking scales were significantly higher and lower, respectively, compared to the non-clinical group (*p* < .0001). The scores on the GPTS and R-GPTS were significantly higher in the clinical group compared to the non-clinical group (*p* < .0001) (Supplementary Table 5). There was nevertheless considerable overlap in the range of the scores between the non-clinical and clinical group.

Supplementary Table 3. Associations between the 10 item FaST questionnaire and the Revised Green et al. Paranoid Thought Scale in the non-clinical group (n=209).

|  | FaST^FAST^ | FaST^SLOW^ | GPTS^TOTAL^ | GPTS^REF^ | GPTS^PERS^ | |
| --- | --- | --- | --- | --- | --- | --- |
| FaST^FAST^ | 1 |  |  |  |  |  |
| FaST^SLOW^ | -.16* | 1 |  |  |  |  |
| R-GPTS^TOTAL^ | .36** | -.12 | 1 |  |  |  |
| R-GPTS^REF^ | .28** | -.14* | .91** | 1 |  |  |
| R-GPTS^PERS^ | .36** | -.06 | .90** | .63** | 1 |  |

* p = < .05

** p = < .01

Supplementary Table 4. Associations between the 10 item FaST questionnaire and the Revised Green et al. Paranoid Thought Scale in the clinical group (n=265).

|  | FaST^FAST^ | FaST^SLOW^ | GPTS^TOTAL^ | GPTS^REF^ | GPTS^PERS^ | |
| --- | --- | --- | --- | --- | --- | --- |
| FaST^FAST^ | 1 |  |  |  |  |  |
| FaST^SLOW^ | -.05 | 1 |  |  |  |  |
| R-GPTS^TOTAL^ | .39** | .16* | 1 |  |  |  |
| R-GPTS^REF^ | .28** | .16** | .87** | 1 |  |  |
| R-GPTS^PERS^ | .40** | .12 | .89** | .54** | 1 |  |

* p = < .05

** p = < .01

Supplementary Table 5. Comparison between clinical and non-clinical groups on the FaST questionnaire, Green et al. Paranoid Thought Scale (GPTS) and Revised-GPTS.

|  | Mean (S.D) | | t | Range | |
| --- | --- | --- | --- | --- | --- |
|  | Non-clinical  group (n = 209) | Clinical group  (n = 265) |  | Non-clinical group  (n = 209) | Clinical group  (n = 265) |
| FaST^FAST^ | 10.6 (4.3) | 16.9 (4.5)** | 15.3 | 5-25 | 6-25 |
| FaST^SLOW^ | 18.9 (4.2) | 17.0 (4.4)** | -4.9 | 7-25 | 5-25 |
| GPTS^TOTAL^ | 51.8 (21.2) | 106.9 (26.0)** | 26.7 | 32-160 | 54-160 |
| GPTS^REF^ | 29.5 (12.7) | 50.1 (15.3)** | 16.9 | 16-80 | 16-80 |
| GPTS^PERS^ | 22.4 (10.5) | 56.7 (13.7)** | 32.7 | 16-80 | 30-80 |
| R-GPTS^TOTAL^ | 13.4 (12.9) | 41.3 (14.6)** | 22.0 | 0-72 | 11-72 |
| R-GPTS^REF^ | 8.9 (7.3) | 16.6 (8.0)** | 10.9 | 0-32 | 0-32 |
| R-GPTS^PERS^ | 4.5 (6.9) | 24.7 (8.8)** | 27.2 | 0-40 | 7-40 |

***p* = < .01
